# LncRNA DLX6-AS1 aggravates the development of ovarian cancer via modulating FHL2 by sponging miR-195-5p

**DOI:** 10.1186/s12935-020-01452-z

**Published:** 2020-08-05

**Authors:** Lijun Kong, Chengyan Zhang

**Affiliations:** Department of Obstetrics and Gynecology, Beijing Obstetrics and Gynecology Hospital, Capital Medical University, No. 251 Yaojiayuan Road, Chaoyang District, Beijing, 100026 China

**Keywords:** DLX6-AS1, Ovarian cancer, miR-195-5p, FHL2, Cancer development

## Abstract

**Background:**

Ovarian cancer (OC) is a huge burden on women’s lives. Recently, the implication of long non-coding RNAs (lncRNAs) in cancers, including OC, has aroused much attention. The objective of this study was to explore the role and functional mechanism of lncRNA distal-less homeobox 6 antisense 1 (DLX6-AS1) in OC.

**Methods:**

The expression of DLX6-AS1, miR-195-5p, and four and a half LIM domains protein 2 (FHL2) was measured by quantitative real-time polymerase chain reaction (qRT-PCR). The cell proliferation, apoptosis, migration, and invasion were assessed by cell count kit 8 (CCK-8), flow cytometry and transwell assays, respectively. The protein levels of proliferating cell nuclear antigen (PCNA), cleaved-caspase-3 (C-caspase 3), N-cadherin, Vimentin, E-cadherin and FHL2 were quantified by western blot. The relationship between miR-195-5p and DLX6-AS1 or FHL2 was predicted by bioinformatics tool starBase and verified by luciferase reporter assay and RNA immunoprecipitation (RIP) assay. Xenograft tumor model was established to observe the role of DLX6-AS1 in vivo.

**Results:**

DLX6-AS1 and FHL2 were up-regulated in OC tissues and cells, while miR-195-5p was down-regulated. DLX6-AS1 knockdown inhibited proliferation, migration, and invasion but induced apoptosis of OC cells. However, miR-195-5p inhibition reversed these effects. Overexpression of miR-195-5p also depleted proliferation, migration, and invasion but promoted apoptosis of OC cells, while FHL2 overexpression overturned these influences. DLX6-AS1 knockdown blocked tumor growth in vivo.

**Conclusion:**

DLX6-AS1, as an oncogene in OC, accelerated tumor progression by up-regulating FHL2 via mediating miR-195-5p, suggesting that DLX6-AS1 was a hopeful target for the lncRNA-targeted therapy in OC.

## Highlights

1. DLX6-AS1 is up-regulated in OC tissues and cells.
2. DLX6-AS1 knockdown attenuates proliferation, migration and invasion but promoted apoptosis of OC cells.
3. DLX6-AS1 exerts its role in OC via the miR-195-5p/FHL2 axis.
4. DLX6-AS1 impedes tumor growth in vivo.

## Background

Ovarian cancer (OC), a common gynecological malignant tumor, is the fifth primary cause of death among women [1, 2]. There are more than 230,000 newly diagnosed cases each year [3]. Unfortunately, most patients diagnosed with OC are already in the advanced stage because of the atypical or asymptomatic nature in the early stage [4]. OC is highly heterogeneous and metastatic [5, 6]. Although the patients respond well at the beginning of chemotherapy, the patients are prone to chemotherapy resistance and recurrence in the advanced stage, resulting in an unfavorable prognosis [7]. Therefore, the exploration of the mechanism of OC development and the identification of related biomarkers are important for the treatment of OC.

Long non-coding RNAs (lncRNAs), over 200 nucleotides in length, have been identified and described a lot with the development of RNA-seq technologies and bioinformatics technologies [8]. Recently, accumulating evidence proved that dysregulation of lncRNAs was implicated in the tumorigenesis and progression [9, 10], including OC. For example, lncRNA NORAD was up-regulated in epithelial OC, and its knockdown efficiently reduced the malignant activities of OC cells [11]. LncRNA UCA1 could enhance the propagation of oncolytic vaccinia virus, leading to a better outcome of OC treatment [12]. LncRNA MALAT1 abnormal overexpression was concerned with the increased stage, recurrence, and poor prognosis in OC [13]. These findings indicated that the pathological processes of OC were connected with abnormal regulation of multiple lncRNAs. Distal-less homeobox 6 antisense 1 (DLX6-AS1) was reported to promote the development of numerous cancers, such as liver cancer [14], osteosarcoma [15] and non-small cell lung cancer [16]. Unfortunately, the literature of DLX6-AS1 in OC is limited, and the detailed function and regulatory mechanism of DLX6-AS1 in OC need further exploring.

MicroRNAs (miRNAs), 18–22 nucleotides in length, are a cluster of non-coding RNAs [17]. Generally speaking, miRNAs play their functions by directing the miRNA-induced silencing complex to target mRNAs, conducing to the inhibition of gene expression at the post-transcriptional level [18]. It was well documented that miRNAs regulated multiple processes in cancer, such as invasion, metastasis, tumor angiogenesis, and inflammation [18]. Interestingly, miR-195-5p was characterized to participate in the inhibition of cancer progression. Acting as a tumor suppressor, miR-195-5p blocked cancer cells migration, invasion, and proliferation in gastric cancer [19], colorectal cancer [20] and thyroid cancer [21]. However, the role of miR-195-5p in OC is not fully elucidated, and the interaction between miR-195-5p and DLX6-AS1 is unknown.

Four and a half LIM domains protein 2 (FHL2) is a member of the four-and-a-half-LIM-only protein family. FHL2 functioned in multiple modes of action, serving as a coactivator of the androgen receptor [22], interacting with β-catenin [23] or acting as a regulator of multiple genes [24]. Recently, the role of FHL2 in the activity of cancers has been mentioned a lot. By and large, high expression of FHL2 was mainly associated with tumorigenesis, aggravation and poor prognosis in cervical cancer and acute myeloid leukemia [25, 26]. Nevertheless, the expression and biological role of FHL2 in OC are vague.

The objective of this study was to explore the role and underlying functional mechanism of DLX6-AS1 in OC. The results of our paper showed a novel mechanism of DLX6-AS1/miR-195-5p/FHL2 axis in OC development and provided a theoretical target for the treatment of OC.

## Materials and methods

### OC tumor tissues

Our research was approved by the Ethics Committee of Beijing Obstetrics and Gynecology Hospital, Capital Medical University. A sum of 50 OC tissues and adjacent healthy tissues were acquired from Beijing Obstetrics and Gynecology Hospital, Capital Medical University. These tissues were swiftly frozen by liquid nitrogen and later placed in − 80 ºC conditions. Each patient signed the informed consent before the clinical operation. The clinicopathologic characteristics of OC patients were presented in Table 1.

**Table 1:** Correlation between DLX6-AS1 expression and clinicopathological parameters of patients with ovarian cancer

Clinical feature	n	DLX6-AS1		*P* value
		High	Low	
Age (years)				0.3943
≥ 50	27	12	15	
< 50	23	13	10	
CA-125 (U/mL)				0.5688
≥ 500	28	13	15	
< 500	22	12	10	
Histological grade				*0.0209*
G1-2	30	11	19	
G3	20	14	6	
FIGO				*0.0087*
III + IV	19	14	5	
I + II	31	11	20	
Lymph node metastasis				*0.0055*
Negative	35	13	22	
Positive	15	12	3	

*P* value < 0.05 indicates significant difference

### Cell lines and culture

OC cell lines (SKOV3 and A2780), normal ovarian epithelial cell line (IOSE80), and human embryonic kidney cell (293 T) were obtained from BeNa Culture Collection (Suzhou, China). Based on the direction, A2780, IOSE80 and 293 T were kept in 90% Dulbecco’s modified Eagle’s medium (DMEM; Gibco, Grand Island, NY, USA) with 10% fetal Bovine Serum (FBS; Gibco). SKOV3 was cultured in 90% Roswell Park Memorial Institute 1640 (RPMI 1640; Gibco) containing 10% FBS (Gibco). Cell cultures were placed in 37℃ conditions with 5% CO_2_.

### Quantitative real-time polymerase chain reaction (qRT-PCR)

Total RNA was separated from tissues (OC tissues and normal tissues) and cells (SKOV3, A2780, IOSE80 and 293 T) using Total RNA Extractor (Sangon Biotech, Shanghai, China). Then 1 µg total RNA was assembled into complementary DNA (cDNA) using the HiScript III 1st Strand cDNA Synthesis Kit (Vazyme, Nanjing, China) or miRNA 1st Strand cDNA Synthesis Kit (Vazyme) for DLX6-AS1 and FHL2 or miR-195-5p. Next, cDNA was utilized to conduct qRT-PCR analysis using AceQ Universal SYBR qPCR Master Mix (Vazyme) on CFX Connect system (Bio-Rad, Hercules, CA, USA). The fold-change of expression was analyzed using the 2^−ΔΔCt^ method. Glyceraldehyde-3-phosphate dehydrogenase (GAPDH) was used as the internal reference for DLX6-AS1 and FHL2, and small nuclear RNA U6 was used as the internal reference for miR-195-5p. The relevant primers were displayed as below: DLX6-AS1, forward (F): 5′-AGTTTCTCTCTAGATTGCCTT-3′ and reverse (R): 5′-ATTGACATGTTAGTGCCCTT-3′; FHL2, F: 5′-GCCAACACCTGCGAGGAGT-3′ and R: 5′-AGTGCCGGTCCTTGTAAGACA-3′; GAPDH, F: 5′-ACCACAGTCCATGCCATCAC-3′ and R: 5′TCCACCACCCT GTTGCTGTA-3′. MiR-195-5p, F: 5′-CGGGATCCACATCTGGGGCCTTGTGA-3′ and R: 5′-CCCAAGCTTGCTTCGTGCTGTCTGCTT-3′. U6, F: 5′-GCUUCGGCAGCACAUAUACUAAAAU-3′ and R: 5′-CGCUUCACGAAUUUGCGUGUCAU-3′.

### Cell transfection

Small interference RNA against DLX6-AS1 (si-DLX6-AS1) and its negative control (si-NC) were synthesized by Sangon Biotech. MiR-195-5p mimic (miR-195-5p; catalog number: miR10000461-1-5) or miR-195-5p inhibitor (anti-miR-195-5p; catalog number: miR20000461-1-5) together with negative control (NC or anti-NC) were purchased from Ribobio (Guangzhou, China). For DLX6-AS1 and FHL2 overexpression, pcDNA3.1 containing DLX6-AS1 sequences (pcDNA-DLX6-AS1), pcDNA3.1 containing FHL2 sequences (FHL2) and their controls (pcDNA-NC and vector) were constructed by Sangon Biotech. Cell transfection was conducted using Lipofectamine 2000 reagent (Invitrogen, Carlsbad, CA, USA).

### Cell count kit-8 (CCK-8) assay

The OC cells with different transfection were collected and resuspended in corresponding mediums. Then the cells were added into 96-well plates at a density of 5000 cells/well. Later, 10 μL CCK-8 solution (Beyotime, Shanghai, China) was pipetted into each well and the systems were incubated for another 2 h. The absorbance of cells in each well at 450 nm was detected under a microplate reader (Bio-Rad) at a specified period of time (24, 48 and 72 h).

### Flow cytometry assay

The OC cells with different transfection were gathered, rinsed with phosphate buffer saline (PBS), and resuspended in binding buffer (2 × 10^5^ cells/mL) from a Cell Apoptosis Kit (Invitrogen). Whereafter, 5 μL Annexin V-FITC and 10 μL propidium Iodide (PI) (20 µg/mL) were pipetted into 100 μL system, and the system was reacted for 15 min in the dark. Finally, the apoptotic cells were distinguished using flow cytometer S3™ Cell Sorter (Bio-Rad).

### Transwell assay

The cell migration and invasion were observed using 24-well transwell chambers (10 μm pore size; BD Biosciences, San Jose, CA, USA). In brief, the cells were trypsinized in serum-free medium and placed into the upper chambers (1 × 10^5^  cells per well). Meanwhile, RPMI-1640 medium or DMEM medium supplemented with 10% FBS was added into the lower chambers. Particularly, the upper chambers needed to be pre-coated with Matrigel (BD Biosciences) for invasion analysis. After 24 h, the cells in the lower surface were fixed with methanol and stained with 0.1% crystal violet solution. At last, a microscope (Olympus, Tokyo, Japan) was applied to analyze the migrated and invaded cells in 5 randomly selected regions.

### Western blot

Western blot analysis was conducted as previously described [27]. The primary antibodies against proliferating cell nuclear antigen (PCNA; ab29; 1:1000), total-caspase 3 (ab13847; 1:500), cleaved-caspase-3 (C-caspase 3; ab13847; 1:1000), N-cadherin (ab202030; 1:1000), Vimentin (ab193555; 1:1000), E-cadherin (ab1416; 1:1000), FHL2 (ab12327; 1:1000) and GAPDH (ab8245; 1:1000), and the secondary antibodies, horseradish peroxidase (HRP) conjugated goat anti-rabbit (ab205718; 1:5000) and goat anti-mouse (ab205719; 1:5000), were purchased from Abcam (Cambridge, MA, USA).

### Bioinformatics analysis

The online tool starBase (https://starbase.sysu.edu.cn/) was adapted to screen the potential target genes and analyze the binding sites between miR-195-5p (miRNAID: MIMAT0000461) and DLX6-AS1 (GeneID: ENSG00000231764) or FHL2 (GeneID: ENSG00000115641).

### Dual-luciferase reporter assay

The wild-type (wt) sequences of DLX6-AS1 containing the binding sites of miR-195-5p or the mutant (mut) sequences designed according to the wt sequences were amplified and introduced into the PGL3-basic vector (Promega, Madison, WI, USA), named as DLX6-AS1-wt or DLX6-AS1-mut. Similarly, FHL2-wt 3′ untranslated regions (UTR) and FHL2-mut 3′ UTR were also constructed. Then the correctly sequenced fusion plasmids were co-transfected into SKOV3 and A2780 cells with miR-195-5p or anti-miR-195-5p. After transfection for 48 h, the luciferase activity was observed using the Dual-Luciferase Reporter Assay Kit (Promega) by a Modulus Microplate Luminometer (Turner BioSystems Inc., Sunnyvale, CA, USA). Firefly luciferase activity was measured and normalized by the Renilla luciferase activity.

### RNA immunoprecipitation (RIP) assay

The RIP assay was consistent with the description mentioned previously [28]. Imprint® RNA Immunoprecipitation kit (Sigma, St. Louis, MO, USA) was used for RIP assay. Co-precipitated RNAs were verified by qRT-PCR.

### Xenograft tumor model in vivo

Animal experiments were approved by the Animal Care and Use Committee of Beijing Obstetrics and Gynecology Hospital, Capital Medical University. Briefly, short hairpin RNA (shRNA) lentiviral vector targeting DLX6-AS1 (sh-DLX6-AS1: 5′-GGTTCAGTATAGATTTCTA-3′) and its control (sh-NC: 5′-AATTCTCCGAACGTGTCACGT-3′) were obtained from Sangon Biotech and constructed into lentiviral vector. 293FT packaging cells were added with sh-DLX6-AS1- or sh-NC-lentiviral plasmid, trans-lentiviral packaging mix and Lipofectamine 2000. After maintaining for 24 h, serum-free medium was replaced with culture medium containing 10% FBS, and viral supernatants were collected after another 24 h for cell infection. A2780 cells were incubated with viral supernatants supplemented with 6 μg/mL polybrene for continuing 24 h and then exposed to puromycin for resistance selection. BALB/c mice (six-week-old, female) were obtained from SLAC Laboratory Animal Co., Ltd (Shanghai, China). The mice, split into 2 groups (n = 5), were maintained and treated at a normative condition. A2780 cells (1 × 10^7^ cells) containing sh-DLX6-AS1 or sh-NC were subcutaneously inoculated into the right rib side of mice. The tumors were observed per week and measured using a caliper (volume = length × width^2^/2). After 35 days, the mice were euthanized and the tumors were removed for other analyses.

### Statistical analysis

All data were processed using GraphPad Prism 7 (GraphPad Inc., La Jolla, CA, USA) from over 3 independent repeats, and presented as the mean ± standard deviation (SD). Student’s *t*-test or one-way analysis of variance (ANOVA) with Tukey post doc analysis was used to evaluate differences between two groups or among multiple groups. Spearman’s correlation analysis was used to confirm the correlation between the expression of miR-195-5p and DLX6-AS1 or FHL2. *P* values less than 0.05 were considered significant.

## Results

### DLX6-AS1 was up-regulated in OC tissues and cell lines

**Fig. 1:**
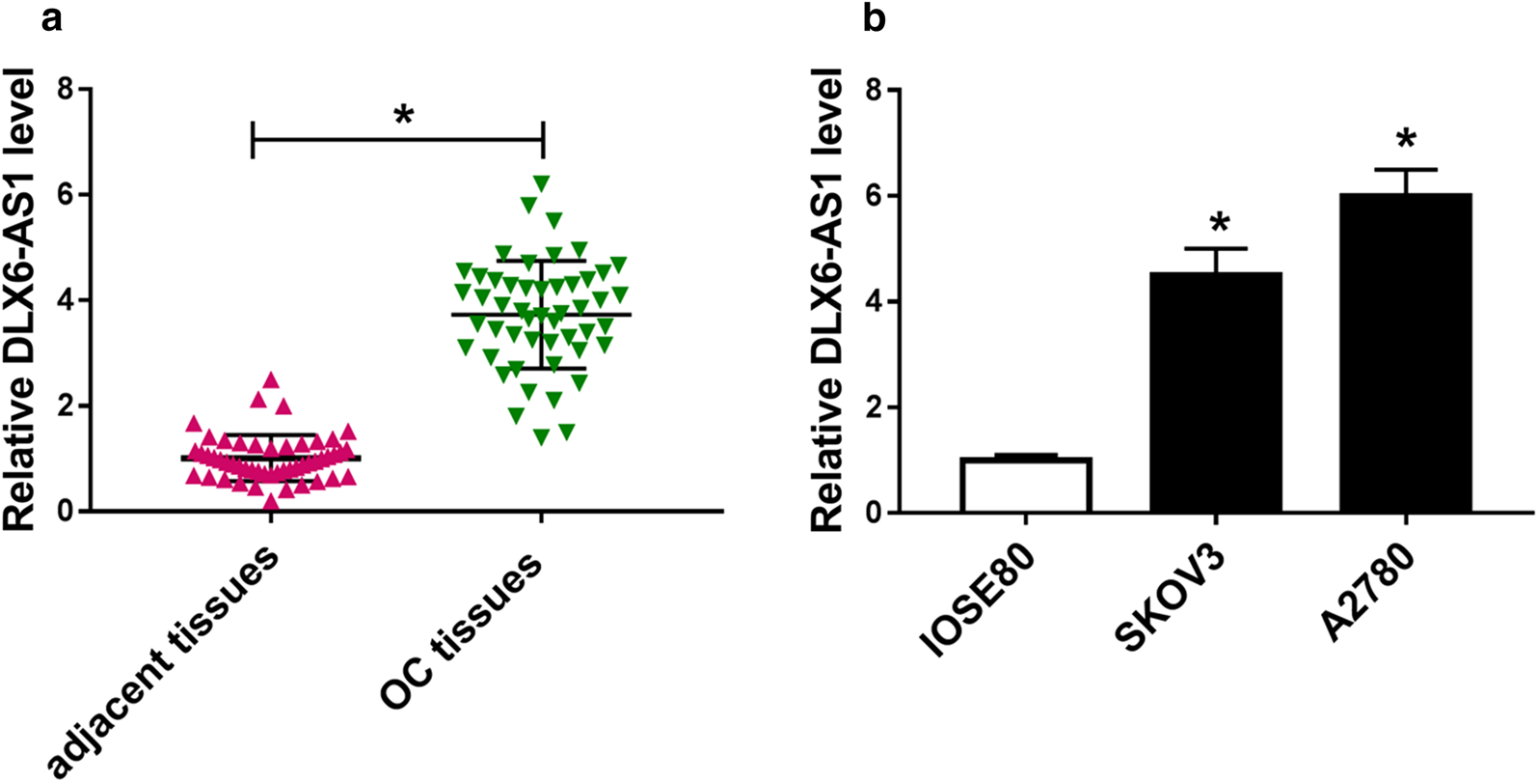
DLX6-AS1 was up-regulated in OC tissues and cell lines. **a** The expression of DLX6-AS1 in OC tissues (n = 50) and adjacent healthy tissues (n = 50) was measured by qRT-PCR. **b** The expression of DLX6-AS1 in OC cell lines (SKOV3 and A2780) and normal ovarian epithelial cell line (IOSE80) was detected by qRT-PCR. *P < 0.05

To monitor the level of DLX6-AS1 in OC, the expression of DLX6-AS1 was measured in OC tissues and cell lines by qRT-PCR. Noticeably, DLX6-AS1 was highly expressed in OC tissues (n = 50) compared with that in adjacent healthy tissues (n = 50) (Fig. 1a). Also, DLX6-AS1 was significantly up-regulated in OC cell lines (SKOV3 and A2780) relative to normal ovarian epithelial cells (IOSE80) (Fig. 1b). The data showed that the dysregulation of DLX6-AS1 might be involved in OC development.

### DLX6-AS1 knockdown inhibited proliferation, migration and invasion but induced apoptosis of OC cells

**Fig. 2:**
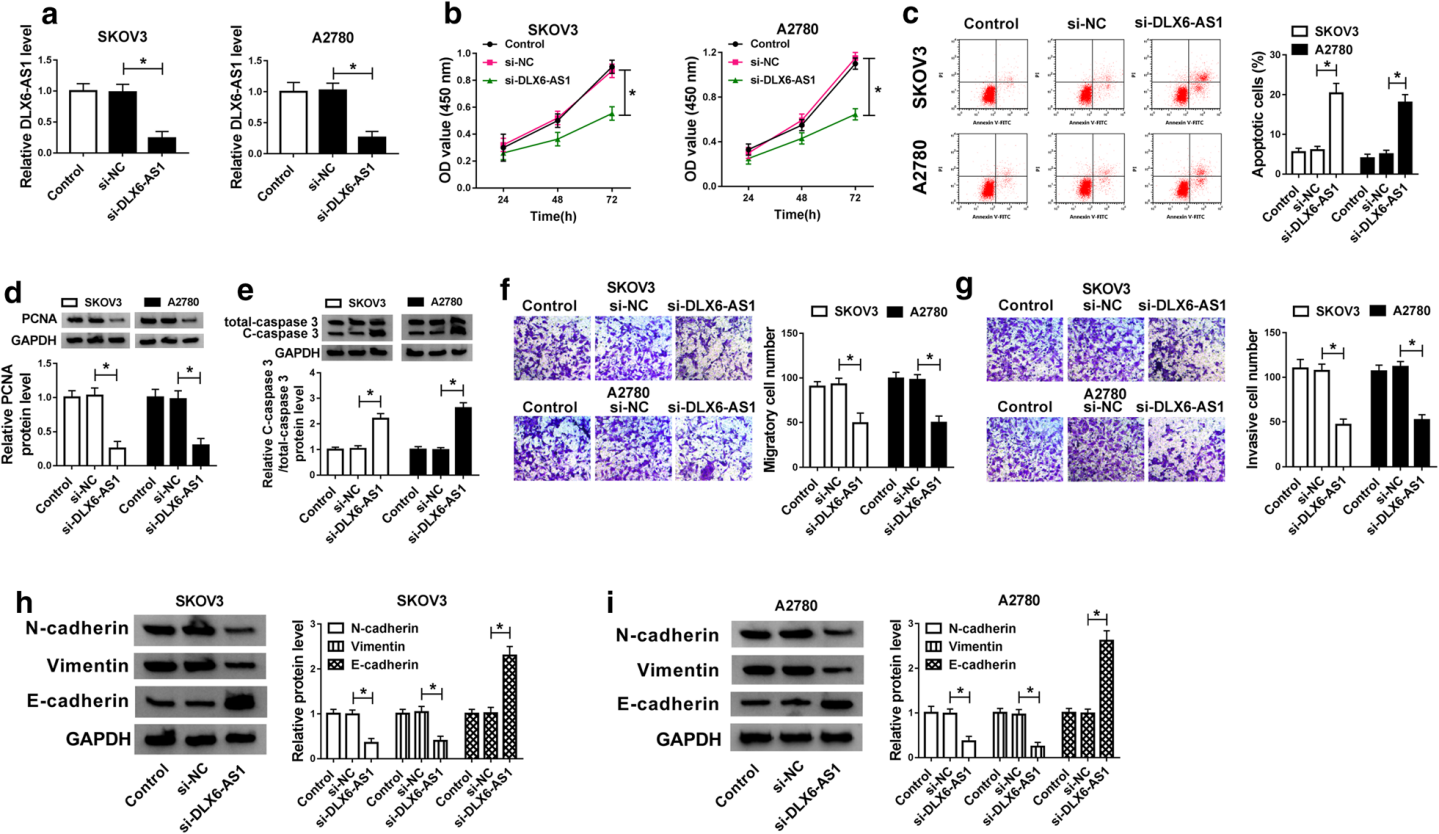
DLX6-AS1 knockdown inhibited OC cells proliferation, migration, and invasion but induced apoptosis. SKOV3 and A2780 cells were transfected with si-DLX6-AS1, with si-NC as a control. **a** The efficiency of DLX6-AS1 knockdown was checked by qRT-PCR in transfected cells. **b** Cell proliferation was assessed by CCK-8 assay in OC cells with DLX6-AS1 knockdown. **c** Cell apoptosis was monitored by flow cytometry assay in OC cells with DLX6-AS1 knockdown. **d**, **e** The protein levels of PCNA and C-caspase 3 in OC cells with DLX6-AS1 knockdown were quantified by western blot analysis. **f**, **g** Cell migration and invasion were detected by transwell assay in OC cells with DLX6-AS1 knockdown. **h**, **i** The protein levels of N-cadherin, Vimentin and E-cadherin in OC cells with DLX6-AS1 knockdown were quantified by western blot analysis. *P < 0.05

To observe the potential role of DLX6-AS1 in OC cells, the endogenous expression level of DLX6-AS1 was knocked down with si-DLX6-AS1 transfection. Firstly, we examined the efficiency of DLX6-AS1 knockdown, and the analysis indicated that the expression of DLX6-AS1 was remarkably decreased in SKOV3 and A2780 cells transfected with si-DLX6-AS1 (Fig. 2a). Next, CCK-8 assay revealed that DLX6-AS1 knockdown significantly declined the ability of proliferation in OC cells (Fig. 2b). Flow cytometry assay presented that the number of apoptotic cells was rapidly increased once DLX6-AS1 was down-regulated in SKOV3 and A2780 cells (Fig. 2c). To further verify the above effects, the expression of PCNA and C-caspase 3 at the protein level was detected. As shown in Fig. 2d, e, the expression of PCNA was markedly reduced, while C-caspase 3 expression was inversely enhanced in SKOV3 and A2780 cells transfected with si-DLX6-AS1. Transwell assay concluded that the number of migratory and invasive cells was collectively weakened in OC cells transfected with si-DLX6-AS1 (Fig. 2f, g). Besides, the protein levels of N-cadherin, Vimentin and E-cadherin were quantified by western blot to check the consequence of migration and invasion. As depicted in Fig. 2h, i, the expression of N-cadherin and Vimentin was notably declined with the decrease of DLX6-AS1 expression, while the expression of E-cadherin strikingly grew. These data manifested that DLX6-AS1 knockdown alleviated the malignant behaviors of OC cells.

### MiR-195-5p was down-regulated in OC cells, and it was a target of DLX6-AS1

**Fig. 3:**
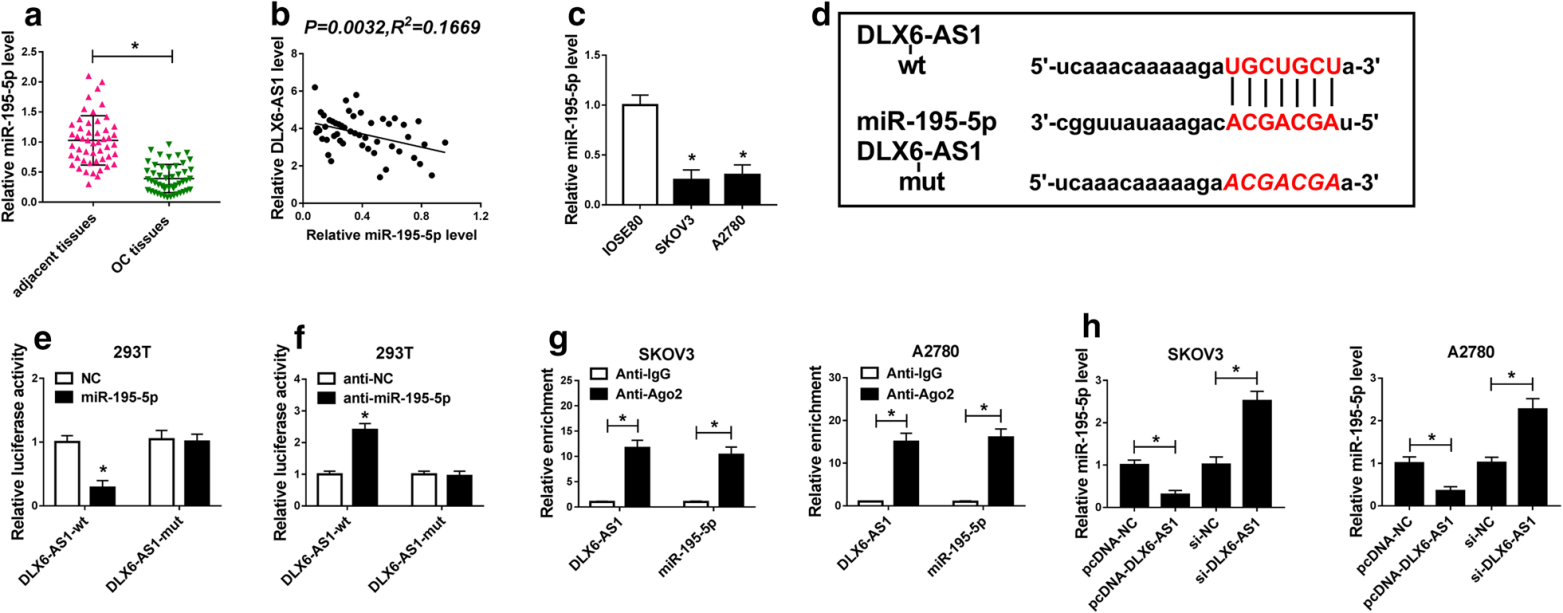
MiR-195-5p was a target of DLX6-AS1 and its expression was declined in OC tissues and cell lines. **a** The expression of miR-195-5p in OC tissues and adjacent healthy tissues was measured by qRT-PCR. **b** Spearman’s correlation analysis revealed the correlation between DLX6-AS1 level and miR-195-5p level in OC tissues. **c** The expression of miR-195-5p in IOSE80, SKOV3 and A2780 was measured by qRT-PCR. **d** Bioinformatics tool starBase analyzed the binding sites between DLX6-AS1 and miR-195-5p. **e**, **f** The relationship between DLX6-AS1 and miR-195-5p was confirmed by dual-luciferase reporter assay. **g** The interaction between DLX6-AS1 and miR-195-5p was further confirmed by RIP assay. (H) The change of miR-195-5p expression level was detected by qRT-PCR in SKOV3 and A2780 cells with DLX6-AS1 overexpression or knockdown. *P < 0.05

To explore the action mechanism of DLX6-AS1 in OC cells, we screened and identified the target miRNA of DLX6-AS1. As exhibited in Fig. 3a, the expression of miR-195a-5p was prominently reduced in OC tissues compared with that in adjacent healthy tissues. Spearman’s correlation analysis revealed that DLX6-AS1 level was negatively correlated with miR-195-5p level in OC tissues (Fig. 3b). Also, the expression of miR-195-5p was declined in OC cell lines (SKOV3 and A2780) relative to normal ovarian epithelial cells (IOSE80) (Fig. 3c). Bioinformatics tool starBase analyzed several binding sites between DLX6-AS1 and miR-195-5p (Fig. 3d). To confirm the relationship between them, dual-luciferase reporter assay was conducted. The result suggested that the transfection with miR-195-5p predominantly decreased the luciferase activity in 293 T cells transfected with DLX6-AS1-wt, but did not alter the luciferase activity in 293 T cells transfected with DLX6-AS1-mut (Fig. 3e). On the contrary, anti-miR-195-5p transfection significantly increased the luciferase activity in cells transfected with DLX6-AS1-wt but did not change the luciferase activity in 293 T cells transfected with DLX6-AS1-mut (Fig. 3f). To further verify the direct interaction between DLX6-AS1 and miR-195-5p in OC cells, RIP assay was performed in SKOV3 and A2780 cells. The result showed that DLX6-AS1 and miR-195-5p were considerably enriched in Ago2 pellets compared with the IgG control group (Fig. 3g). Additionally, DLX6-AS1 overexpression conduced to an apparent decrease of miR-195-5p expression, while DLX6-AS1 knockdown significantly reinforced the expression of miR-195-5p in both SKOV3 and A2780 cells (Fig. 3h). These data indicated that miR-195-5p was indeed a target of DLX6-AS1, and DLX6-AS1 regulated its expression level in OC cells.

### MiR-195-5p inhibition reversed the influence of DLX6-AS1 knockdown on proliferation, apoptosis, migration and invasion in OC cells

**Fig. 4:**
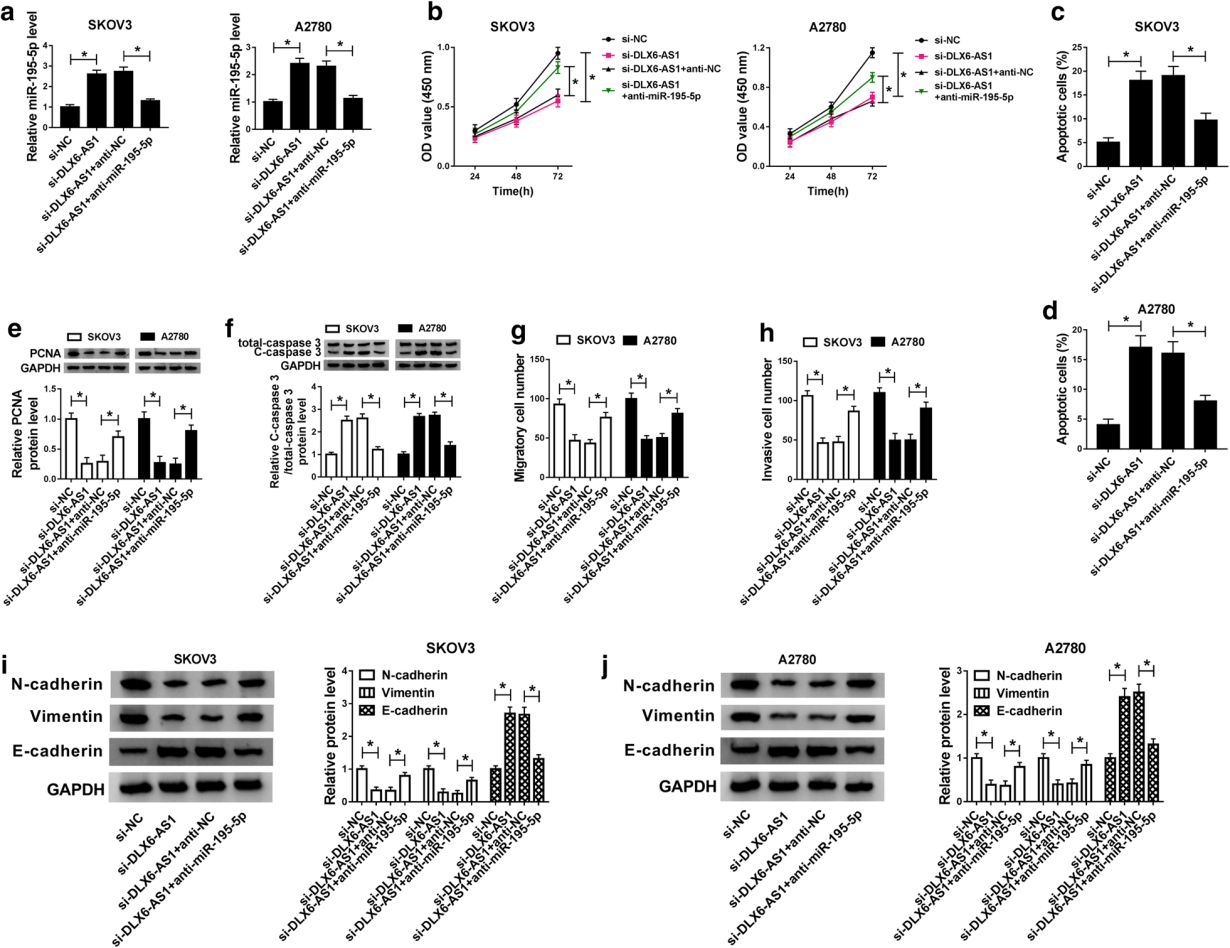
MiR-195-5p inhibition reversed the effects of DLX6-AS1 knockdown on OC cells proliferation, apoptosis, migration and invasion. SKOV3 and A2780 cells were transfected with si-DLX6-AS1, si-NC, si-DLX6-AS1 + anti-miR-195-5p and si-DLX6-AS1 + anti-NC, respectively. **a** The expression level of miR-195-5p was measured by qRT-PCR in indicated cells. **b** Cell proliferation was assessed by CCK-8 assay in indicated cells. **c**, **d** Cell apoptosis was monitored by flow cytometry assay in indicated cells. **e**, **f** The protein levels of PCNA and C-caspase 3 in indicated cells were quantified by western blot analysis. **g**, **h** Cell migration and invasion were detected by transwell assay in indicated cells. **i**, **j** The protein levels of N-cadherin, Vimentin and E-cadherin in indicated cells were quantified by western blot analysis. *P < 0.05

To observe whether DLX6-AS1 functioning by mediating miR-195-5p, SKOV3 and A2780 cells were transfected with si-DLX6-AS1, si-NC, si-DLX6-AS1 + anti-miR-195-5p or si-DLX6-AS1 + anti-NC, respectively. The enhanced level of miR-195-5p caused by si-DLX6-AS1 transfection was swiftly inhibited by si-DLX6-AS1 + anti-miR-195-5p transfection in both SKOV3 and A2780 cells (Fig. 4a). The CCK-8 assay exhibited that inhibition of miR-195-5p promoted proliferation of cells suppressed by DLX6-AS1 knockdown (Fig. 4b). The flow cytometry assay alleged that miR-195-5p inhibition blocked apoptosis of cells induced by DLX6-AS1 knockdown in both SKOV3 and A2780 cells (Fig. 4c, d), and the representative flow cytometry plots were exhibited in Additional file 1. Figure S4c, d. To determine the result of proliferation and apoptosis, we investigated the protein levels of PCNA and C-caspase 3 and found the level of PCNA was reduced in OC cells transfected with si-DLX6-AS1, while si-DLX6-AS1 + anti-miR-195-5p transfection notably strengthened the level of PCNA (Fig. 4e). On the contrary, the level of C-caspase 3 was opposite to that of the PCNA level (Fig. 4f). The transwell assay asserted that si-DLX6-AS1 + anti-miR-195-5p transfection accelerated the number of migratory and invasive cells inhibited by si-DLX6-AS1 transfection in SKOV3 and A2780 cells (Fig. 4g, h), and the migration and invasion images were exhibited in Additional file 1. Figure S4g, h. To ascertain the result of cell migration and invasion, we assessed the protein levels of N-cadherin, Vimentin and E-cadherin and concluded that the expression of N-cadherin and Vimentin was depleted by si-DLX6-AS1 transfection but rescued by si-DLX6-AS1 + anti-miR-195-5p transfection, while the expression of E-cadherin was stimulated by si-DLX6-AS1 transfection but decayed by si-DLX6-AS1 + anti-miR-195-5p transfection (Fig. 4i, j). The above information implied that DLX6-AS1 knockdown alleviated the malignant behaviors of OC cells by enhancing the expression of miR-195-5p.

### FHL2 was a target of miR-195-5p and expressed with a high level in OC tissues and cells

**Fig. 5:**
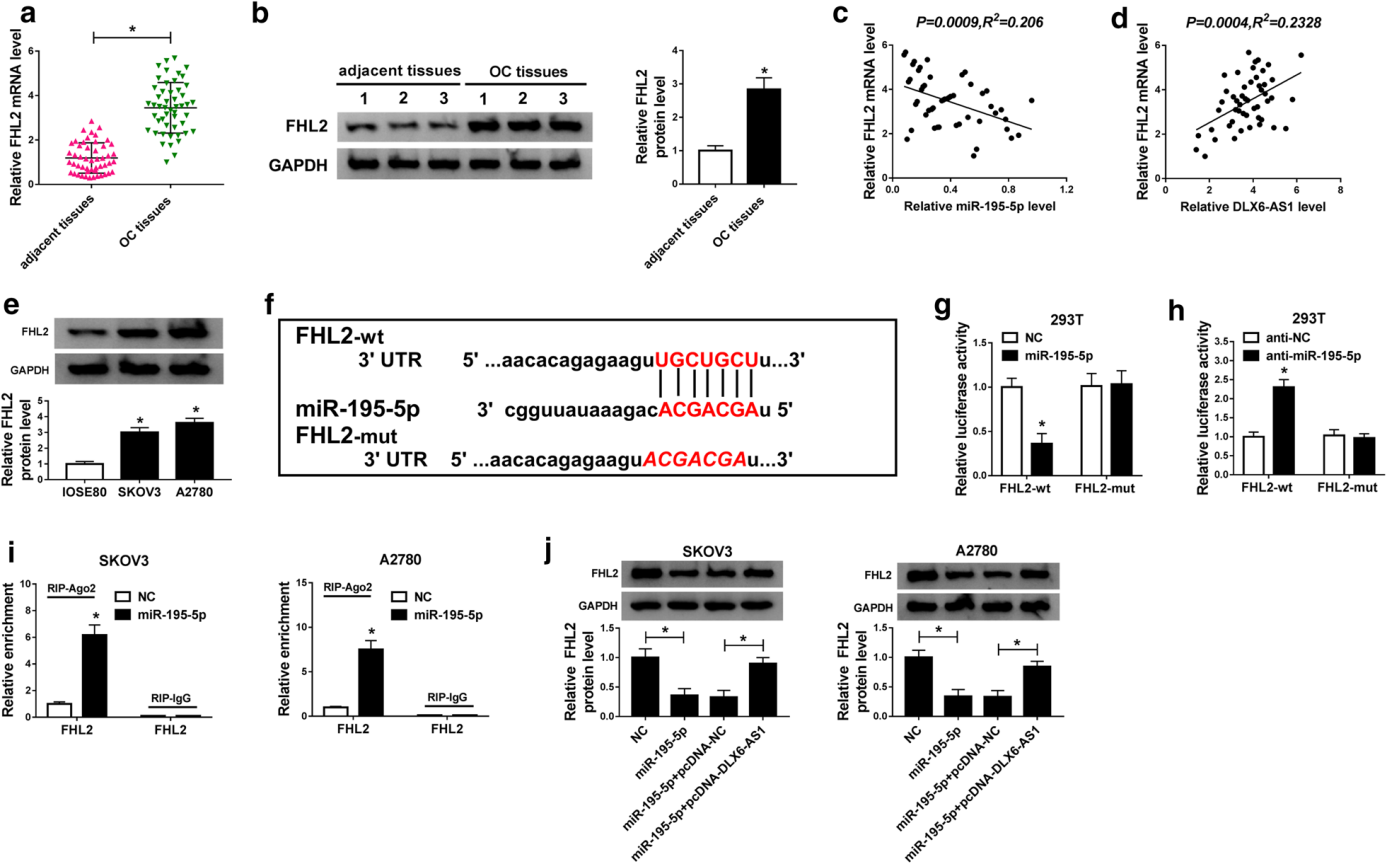
FHL2, up-regulated in OC tissues and cell lines, was a target of miR-195-5p. **a**, **b** The expression of FHL2 at mRNA level and protein level in OC tissues and adjacent healthy tissues was measured by qRT-PCR and western blot, respectively. **c**, **d** Spearman’s correlation analysis revealed the correlation between FHL2 mRNA level and miR-195-5p level or DLX6-AS1 level in OC tissues. **e** The expression of FHL2 in IOSE80, SKOV3 and A2780 was measured by western blot at the protein level. **f** Bioinformatics tool starBase analyzed the binding sites between FHL2 3′ UTR and miR-195-5p. **g**, **h** The relationship between FHL2 and miR-195-5p was confirmed by dual-luciferase reporter assay. **i** The interaction between FHL2 and miR-195-5p was further confirmed by RIP assay. **j** The protein level of FHL2 was detected by western blot in SKOV3 and A2780 cells transfected with miR-195-5p, NC, miR-195-5p + pcDNA-DLX6-AS1 or miR-195-5p + pcDNA-NC. *P < 0.05

To further explore the action mechanism of DLX6-AS1 in OC, the target genes of miR-195-5p were screened and authenticated. Figure 5a, b showed that the expression of FHL2 was significantly up-regulated in OC tissues relative to adjacent healthy tissues at both mRNA and protein levels. Spearman’s correlation analysis pointed out that FHL2 mRNA level was negatively correlated with the miR-195-5p level in OC tissues but positively correlated with the DLX6-AS1 level (Fig. 5c, d). Likewise, the expression of FHL2 at the protein level was increased in OC cell lines (SKOV3 and A2780) relative to normal ovarian epithelial cells (IOSE80) (Fig. 5e). Bioinformatics tool starBase proved that several binding sites existed between FHL2 3′ UTR and miR-195-5p (Fig. 5f). To verify the relationship between them, dual-luciferase reporter assay was performed, and we found the luciferase activity was steeply decreased in 293 T cells transfected with FHL2-wt and miR-195-5p, while the luciferase activity had no visible change in cells transfected with FHL2-mut and miR-195-5p (Fig. 5g). Inversely, the luciferase activity was swiftly enhanced in 293 T cells transfected with FHL2-wt and anti-miR-195-5p, while the luciferase activity had no difference in cells transfected with FHL2-mut and anti-miR-195-5p (Fig. 5h). To further verify the interaction between FHL2 and miR-195-5p, the result from RIP assay showed that miR-195-5p transfection remarkably intensified the enrichment of FHL2 in Ago2 RIP compared to that in lgG control (Fig. 5i). Moreover, we discovered that the protein level of FHL2 was inhibited in SKOV3 and A2780 cells transfected with miR-195a-5p but strengthened in SKOV3 and A2780 cells transfected with miR-195-5p + pcDNA-DLX6-AS1 (Fig. 5j). Above data elucidated that FHL2 was a direct target of miR-195-5p and miR-195-5p modulated its expression in OC cells.

### FHL2 overexpression overturned the effects of miR-195-5p enrichment on cells proliferation, apoptosis, migration and invasion in OC

**Fig. 6:**
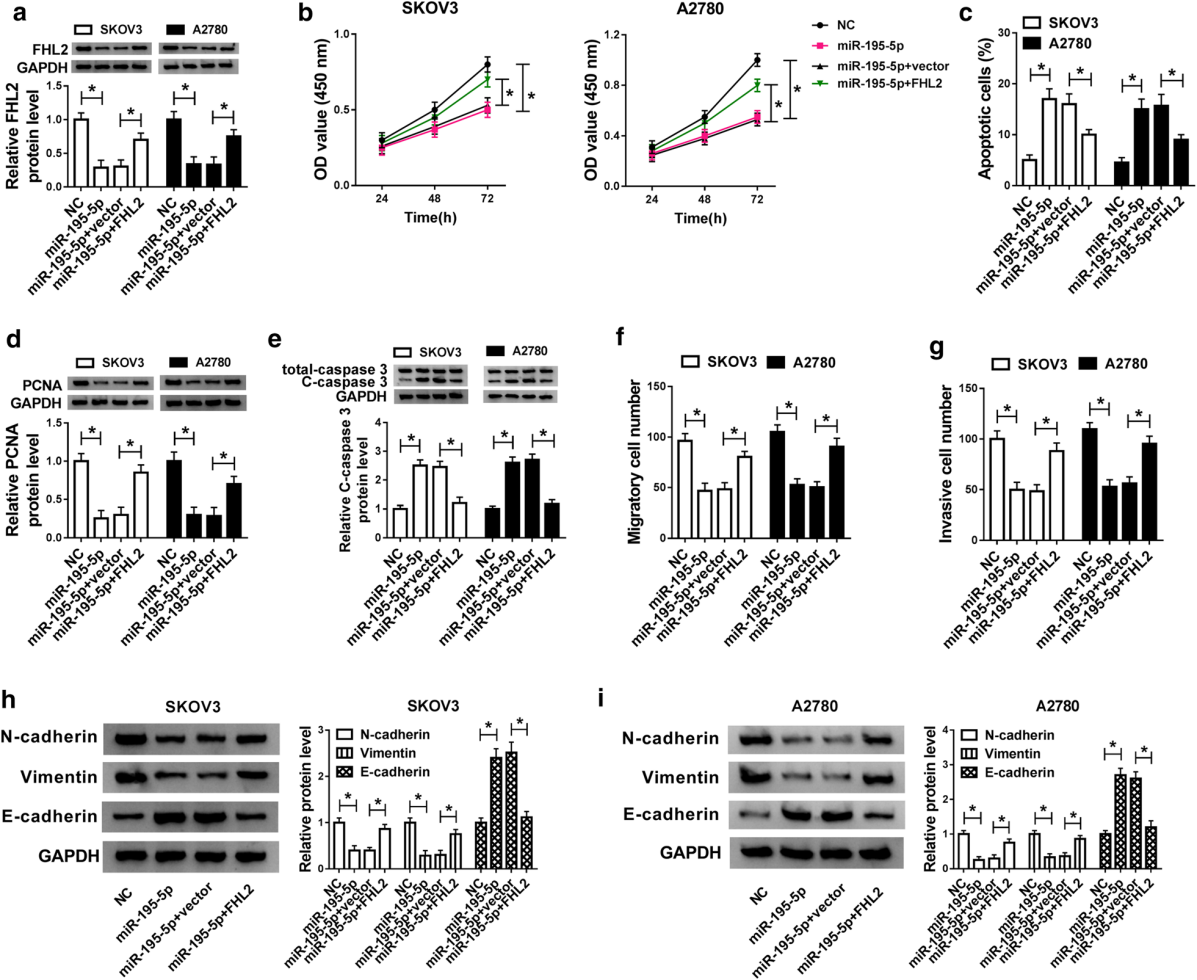
FHL2 overexpression reversed the influences of miR-195-5p enrichment on OC cells proliferation, apoptosis, migration and invasion. SKOV3 and A2780 cells were transfected with miR-195-5p, NC, miR-195-5p + FHL2 and miR-195-5p + vector, respectively. **a** The protein level of FHL2 in mentioned transfected cells was measured by western blot. **b** Cell proliferation was assessed by CCK-8 assay in mentioned transfected cells. **c** Cell apoptosis was monitored by flow cytometry assay in mentioned transfected cells. **d**, **e** The protein levels of PCNA and C-caspase 3 in mentioned transfected cells were quantified by western blot analysis. **f**, **g** Cell migration and invasion were detected by transwell assay in mentioned transfected cells. **h**, **i** The protein levels of N-cadherin, Vimentin and E-cadherin in mentioned transfected cells were quantified by western blot analysis. *P < 0.05

To investigate whether miR-195-5p exerted its role by interacting with FHL2, miR-195-5p, NC, miR-195-5p + FHL2, and miR-195-5p + vector were introduced into SKOV3 and A2780 cells, respectively. The expression of FHL2 at the protein level was suppressed by miR-195-5p transfection but accelerated by miR-195-5p + FHL2 transfection (Fig. 6a). CCK-8 assay described that cell proliferation inhibited by miR-195-5p overexpression was restored by FHL2 synchronous up-regulation in SKOV3 and A2780 cells (Fig. 6b). Flow cytometry assay clarified that the apoptotic rate induced by miR-195-5p overexpression was subdued by FHL2 synchronous up-regulation (Fig. 6c), and the representative flow cytometry plots were displayed in Additional file 2. Figure S6c. Besides, the expression of PCNA at the protein level was inhibited by miR-195-5p transfection but encouraged by miR-195-5p + FHL2 transfection (Fig. 6d), while the expression of C-caspase 3 was opposite to PCNA (Fig. 6e). Next, transwell assay maintained that the number of migratory and invasive cells was declined in cells with miR-195-5p transfection but elevated in cells with miR-195-5p + FHL2 transfection (Fig. 6f, g), and the migration and invasion images were displayed in Additional file 2. Figure S6f, g. Additionally, the protein levels of N-cadherin and Vimentin were dwindled with the increase of miR-195-5p but rescued with the accumulation of FHL2, while the level of E-cadherin was opposite to the levels of N-cadherin and Vimentin in both SKOV3 and A2780 cells (Fig. 6h, i). Altogether, miR-195-5p overexpression blocked the progression of OC cells by inhibiting the expression of FHL2.

### DLX6-AS1 knockdown blocked tumor growth in vivo

**Fig. 7:**
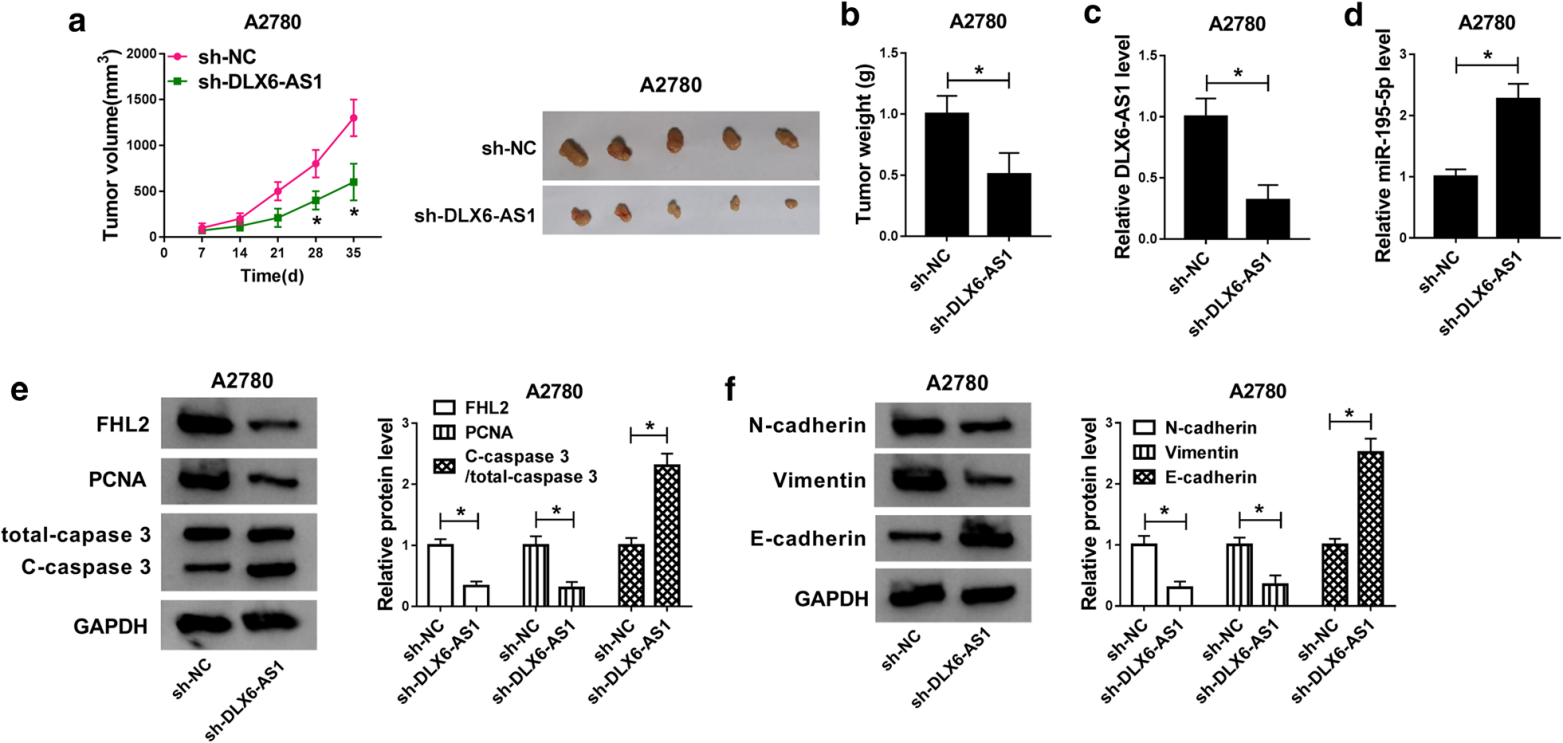
DLX6-AS1 knockdown blocked tumor growth in vivo. **a** The volume of tumors was recorded every 7 days using a caliper. **b** Tumors were weighed after 35 days. **c** DLX6-AS1 level and (**d**) miR-195-5p level were determined by QRT-PCR in removed tissues. **e** The expression of FHL2, PCNA and C-caspase 3 at the protein level was measured by western blot in removed tissues**.f** The expression of N-cadherin, Vimentin and E-cadherin at the protein level was assessed by western blot in removed tissues. *P < 0.05

To determine the role of DLX6-AS1 knockdown in the tumor growth, xenograft model was established. Tumor volume was recorded every 7 days after injection, and the result indicated that DLX6-AS1 knockdown effectively cut the tumor volume down (Fig. 7a). After 35 days, all mice were killed, and the tumor weight was measured. Not surprisingly, tumor weight was also reduced with the injection of sh-DLX6-AS1 relative to sh-NC (Fig. 7b). Then qRT-PCR analysis presented that the expression of DLX6-AS1 was enormously declined in removed tumor tissues from the sh-DLX6-AS1 group compared with the sh-NC group, while miR-195-5p expression was accumulated (Fig. 7c, d). Furthermore, western blot analysis summarized that the expression level of FHL2 was decreased in tumor tissues from the sh-DLX6-AS1 group compared with the sh-NC group. Besides, the protein level of PCNA was reduced in the sh-DLX6-AS1 group, while the expression of C-caspase 3 was enhanced (Fig. 7e). Western blot analysis presented that N-cadherin and Vimentin expression was diminished in tumor tissues from the sh-DLX6-AS1 group, while E-cadherin expression was strengthened (Fig. 7f). Collectively, DLX6-AS1 knockdown inhibited tumor growth in vivo by inducing FHL2 expression via mediating miR-195-5p.

## Discussion

OC is a fearful threat to women’s health and lives. In view of the limitation of conventional treatment, the exploration of novel biomarkers for the therapy of OC is urgent. Our present study found that DLX6-AS1 was up-regulated in OC. In function, DLX6-AS1 knockdown repressed the malignant behaviors of OC cells and inhibited tumor growth in vivo. Mechanically, miR-195-5p was verified as a target of DLX6-AS1, and it also directly interacted with FHL2. Our analyses proved that DLX6-AS1 functioned in OC through modulating FHL2 by sponging miR-195-5p.

DLX6-AS1 exerts its role in the regulation of diverse types of cancer. Illustrating the functions of DLX6-AS1 in the regulation of OC development in a few studies may help to detect novel mechanisms of OC occurrence and metastasis. For example, DLX6-AS1 knockdown inhibited migration and invasion of OC cells, while DLX6-AS1 overexpression contributed to migration and invasion of OC cells by mediating miR-613 [29]. Another report claimed that highly expressed DLX6-AS1 was associated with lymph node metastasis and poor prognosis of OC patients, and downregulation of DLX6-AS1 blocked the expression of Notch1, p21, and Hes1, leading to the suppression of proliferation, migration and invasion in OC cells [30]. These findings highlighted the cancer-promoting role of DLX6-AS1 in OC. Consistent with them, we verified that DLX6-AS1 was up-regulated in OC tissues and cells. Interference of DLX6-AS1 suppressed proliferation, migration and invasion but accelerated apoptosis of OC cells to some extent in vitro. We also performed tumor formation assay in vivo and found that DLX6-AS1 down-regulation decreased tumor growth. All data suggested that DLX6-AS1 was an oncogene in OC.

MiR-195-5p was confirmed as a target of DLX6-AS1. A previous paper stated that the expression of miR-195-5p was declined in OC tissues and miR-195-5p overexpression depleted cisplatin resistance and angiogenesis [31]. The potential functional role of miR-195-5p was obtained from other cancers, such as hepatocellular carcinoma, breast cancer and prostate cancer [32–34]. Collectively, miR-195-5p was consistently down-regulated in these cancers, and its enrichment efficiently suppressed the malignant behaviors of cancer cells. Consistent with the previous studies, our study displayed that miR-195-5p expression was also decreased in OC tissues and cells. Inhibition of miR-195-5p reversed the inhibitory effects on OC development caused by DLX6-AS1 knockdown. Above findings hinted that miR-195-5p was a tumor suppressor in almost all types of cancer, including OC.

FHL2, a target of miR-195-5p, was regulated by DLX6-AS1 through miR-195-5p. FHL2 is a member of LIM domain proteins, which play essential roles in cell growth, cell differentiation, cell cytoskeleton construction and cell fate [35]. Recently, the role of FHL2 in cancers has become increasingly clear, including OC. For instance, FHL2 with high abundance was involved in poor outcome of OC patients and silence of FHL2 suppressed growth and metastasis of OC cells [36]. The study of Gabriel et al. maintained that FHL2 was overexpressed in OC cells [37]. In accordance with these investigations, there was an increased expression of FHL2 in OC tissues and cells. Besides, FHL2 overexpression destroyed the inhibitory development of OC cells triggered by miR-195-5p enrichment, suggesting the tumor promoter role of FHL2 in OC.

In our research, several important marker proteins, such as PCNA, C-caspase 3, N-cadherin, Vimentin and E-cadherin, were detected to validate the change of related cell process. PCNA was a novel endogenous marker for cell proliferation, which was applied in numerous studies to measure cell proliferation [38, 39]. C-caspase 3 was a key protease that indicated cell apoptosis and DNA damage [40]. The activation of EMT was an important program to promote tumor metastasis and invasion [41]. E-cadherin, N-cadherin, and Vimentin were important markers of EMT. Prudkin et al. found that E-cadherin showed low expression, while N-cadherin and Vimentin presented high expression in different pathological types of lung cancer tissues [42]. Similarly in our study, the levels of these markers were monitored to ensure the role of DLX6-AS1, miR-195-5p and FHL2 in proliferation, apoptosis, migration and invasion.

## Conclusion

Taken together, DLX6-AS1 was expressed with a high level in OC tissues and cells. Functional analysis revealed that DLX6-AS1 might serve as a tumor promoter in the development of OC by upregulating FHL2 expression via competitively binding to miR-195-5p in vitro and in vivo. Our research suggests that DLX6-AS1 may be a promising biomarker for the diagnosis and treatment of OC.

## Supplementary information

**Additional file 1: Figure S4** The representative images of (**c**, **d**) apoptosis, (**g**) migration and (**h**) invasion in SKOV3 and A2780 cells transfected with si-DLX6-AS1, si-NC, si-DLX6-AS1+anti-miR-195-5p or si-DLX6-AS1+anti-miR-NC.

**Additional file 2: Figure S6** The representative images of (**c**) apoptosis, (**f**) migration and (**g**) invasion in SKOV3 and A2780 cells transfected with miR-195-5p, NC, miR-195-5p+FHL2 or miR-195-5p+vector.

## Data Availability

The analyzed data sets generated during the present study are available from the corresponding author on reasonable request.

## Abbreviations

OC: Ovarian cancer
qRT-PCR: Quantitative real-time polymerase chain reaction
CCK-8: Cell count kit 8
PCNA: Proliferating cell nuclear antigen
RIP: RNA immunoprecipitation
DLX6-AS1: Distal-less homeobox 6 antisense 1

## References

[1] Cornelison R (2017). Emerging therapeutics to overcome chemoresistance in epithelial ovarian cancer: a mini-review. Int J Mol Sci.

[2] Teo MC (2014). Update on the management and the role of intraperitoneal chemotherapy for ovarian cancer. Curr Opin Obstet Gynecol.

[3] Hassan MK (2011). Intracellular clusterin negatively regulates ovarian chemoresistance: compromised expression sensitizes ovarian cancer cells to paclitaxel. Tumour Biol.

[4] van Jaarsveld MT (2010). MicroRNAs in ovarian cancer biology and therapy resistance. Int J Biochem Cell Biol.

[5] Vergara D (2010). Epithelial-mesenchymal transition in ovarian cancer. Cancer Lett.

[6] Hollis RL (2016). Genetic and molecular changes in ovarian cancer. Cancer Biol Med.

[7] Allemani C (2018). Global surveillance of trends in cancer survival 2000–14 (CONCORD-3): analysis of individual records for 37 513 025 patients diagnosed with one of 18 cancers from 322 population-based registries in 71 countries. Lancet.

[8] Yang G (2014). LncRNA: a link between RNA and cancer. Biochim Biophys Acta.

[9] Jiang MC (2019). Emerging roles of lncRNA in cancer and therapeutic opportunities. Am J Cancer Res.

[10] Ghafouri-Fard S (2019). Long noncoding RNA PVT1: a highly dysregulated gene in malignancy. J Cell Physiol.

[11] Tong L (2019). Long noncoding RNA NORAD is upregulated in epithelial ovarian cancer and its downregulation suppressed cancer cell functions by competing with miR-155–5p. Cancer Med.

[12] Horita K (2019). lncRNA UCA1-mediated Cdc42 signaling promotes oncolytic vaccinia virus cell-to-cell spread in ovarian cancer. Mol Ther Oncolytics.

[13] Gordon MA (2019). The long non-coding RNA MALAT1 promotes ovarian cancer progression by regulating RBFOX2-mediated alternative splicing. Mol Carcinog.

[14] Wu DM (2019). Down-regulated lncRNA DLX6-AS1 inhibits tumorigenesis through STAT3 signaling pathway by suppressing CADM1 promoter methylation in liver cancer stem cells. J Exp Clin Cancer Res.

[15] Zhang N (2019). LncRNA DLX6-AS1 promotes tumor proliferation and metastasis in osteosarcoma through modulating miR-641/HOXA9 signaling pathway. J Cell Biochem.

[16] Huang Y (2019). Knockdown of lncRNA DLX6-AS1 inhibits cell proliferation, migration and invasion while promotes apoptosis by downregulating PRR11 expression and upregulating miR-144 in non-small cell lung cancer. Biomed Pharmacother.

[17] Morishita A (2016). MicroRNA profiles in various hepatocellular carcinoma cell lines. Oncol Lett.

[18] Panoutsopoulou K (2018). miRNA and long non-coding RNA: molecular function and clinical value in breast and ovarian cancers. Expert Rev Mol Diagn.

[19] Zhao DL (2019). Effect of inhibition to Yes-related proteins-mediated Wnt/beta-catenin signaling pathway through miR-195-5p on apoptosis of gastric cancer cells. Eur Rev Med Pharmacol Sci.

[20] Bai J (2019). lncRNA SNHG1 cooperated with miR-497/miR-195-5p to modify epithelial-mesenchymal transition underlying colorectal cancer exacerbation. J Cell Physiol.

[21] Du P (2019). Linc00210 enhances the malignancy of thyroid cancer cells by modulating miR-195-5p/IGF1R/Akt axis. J Cell Physiol.

[22] Muller JM (2000). FHL2, a novel tissue-specific coactivator of the androgen receptor. EMBO J.

[23] Martin B (2002). The LIM-only protein FHL2 interacts with beta-catenin and promotes differentiation of mouse myoblasts. J Cell Biol.

[24] Paul C (2006). The LIM-only protein FHL2 is a negative regulator of E4F1. Oncogene.

[25] Jin X (2018). Increased expression of FHL2 promotes tumorigenesis in cervical cancer and is correlated with poor prognosis. Gene.

[26] Cheng Z (2019). Enhanced expressions of FHL2 and iASPP predict poor prognosis in acute myeloid leukemia. Cancer Gene Ther.

[27] Zhu Y (2016). MicroRNA-217 inhibits cell proliferation and invasion by targeting Runx2 in human glioma. Am J Transl Res.

[28] Feng L (2019). Long noncoding RNA DLEU1 aggravates glioma progression via the miR-421/MEF2D axis. Onco Targets Ther.

[29] You Q (2019). Long non-coding RNA DLX6-AS1 acts as an oncogene by targeting miR-613 in ovarian cancer. Eur Rev Med Pharmacol Sci.

[30] Zhao J (2019). Down-regulation of long noncoding RNA DLX6-AS1 defines good prognosis and inhibits proliferation and metastasis in human epithelial ovarian cancer cells via Notch signaling pathway. Eur Rev Med Pharmacol Sci.

[31] Dai J (2019). Overexpression of microRNA-195-5p reduces cisplatin resistance and angiogenesis in ovarian cancer by inhibiting the PSAT1-dependent GSK3beta/beta-catenin signaling pathway. J Transl Med.

[32] Xu H (2015). MicroRNA-195-5p acts as an anti-oncogene by targeting PHF19 in hepatocellular carcinoma. Oncol Rep.

[33] Luo Q (2014). MicroRNA-195-5p is a potential diagnostic and therapeutic target for breast cancer. Oncol Rep.

[34] Wu J (2015). MicroRNA-195–5p, a new regulator of Fra-1, suppresses the migration and invasion of prostate cancer cells. J Transl Med.

[35] Rafael MS (2012). Four-and-a-half LIM domains protein 2 (FHL2) is associated with the development of craniofacial musculature in the teleost fish Sparus aurata. Cell Mol Life Sci.

[36] Huang Z (2019). miR-340-FHL2 axis inhibits cell growth and metastasis in ovarian cancer. Cell Death Dis.

[37] Gabriel B (2004). Focal adhesion kinase interacts with the transcriptional coactivator FHL2 and both are overexpressed in epithelial ovarian cancer. Anticancer Res.

[38] Dietrich DR (1993). Toxicological and pathological applications of proliferating cell nuclear antigen (PCNA), a novel endogenous marker for cell proliferation. Crit Rev Toxicol.

[39] Xie Y (2018). A 3-Protein Expression Signature of Neuroblastoma for Outcome Prediction. Am J Surg Pathol.

[40] Chua S (2016). The cardioprotective effect of melatonin and exendin-4 treatment in a rat model of cardiorenal syndrome. J Pineal Res.

[41] Wellner U (2009). The EMT-activator ZEB1 promotes tumorigenicity by repressing stemness-inhibiting microRNAs. Nat Cell Biol.

[42] Prudkin L (2009). Epithelial-to-mesenchymal transition in the development and progression of adenocarcinoma and squamous cell carcinoma of the lung. Mod Pathol.

