# Supplementary figures and images for: LncRNA DLX6-AS1 aggravates the development of ovarian cancer via modulating FHL2 by sponging miR-195-5p
Source: Cancer Cell Int. 2020 Aug 5;20:370. doi: 10.1186/s12935-020-01452-z (PMC7405350; doi:10.1186/s12935-020-01452-z)

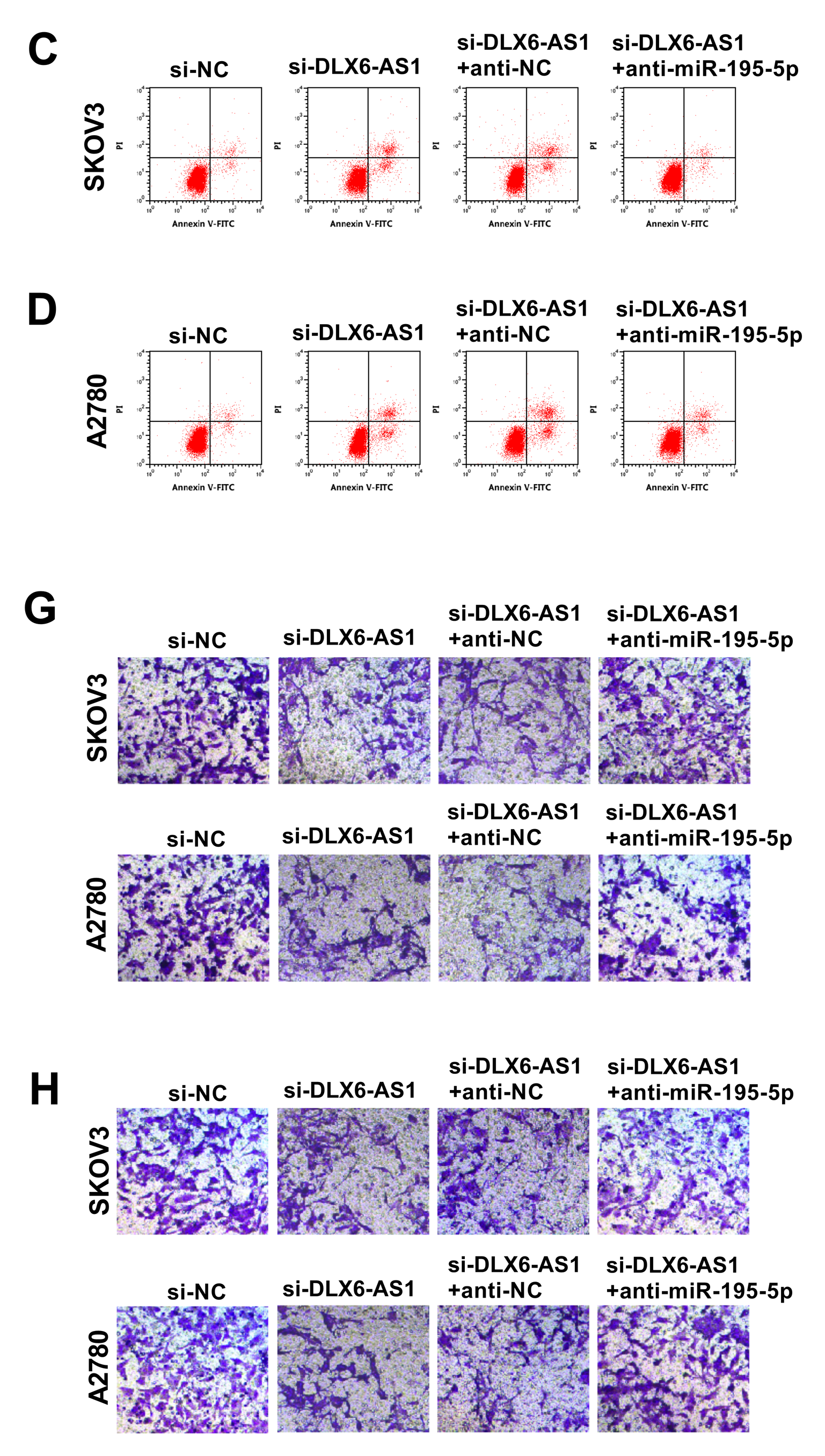

Supplement: Supplementary file 1 — Additional file 1: Figure S4 The representative images of (c, d) apoptosis, (g) migration and (h) invasion in SKOV3 and A2780 cells transfected with si-DLX6-AS1, si-NC, si-DLX6-AS1+anti-miR-195-5p or si-DLX6-AS1+anti-miR-NC. [file 12935_2020_1452_MOESM1_ESM.tif]

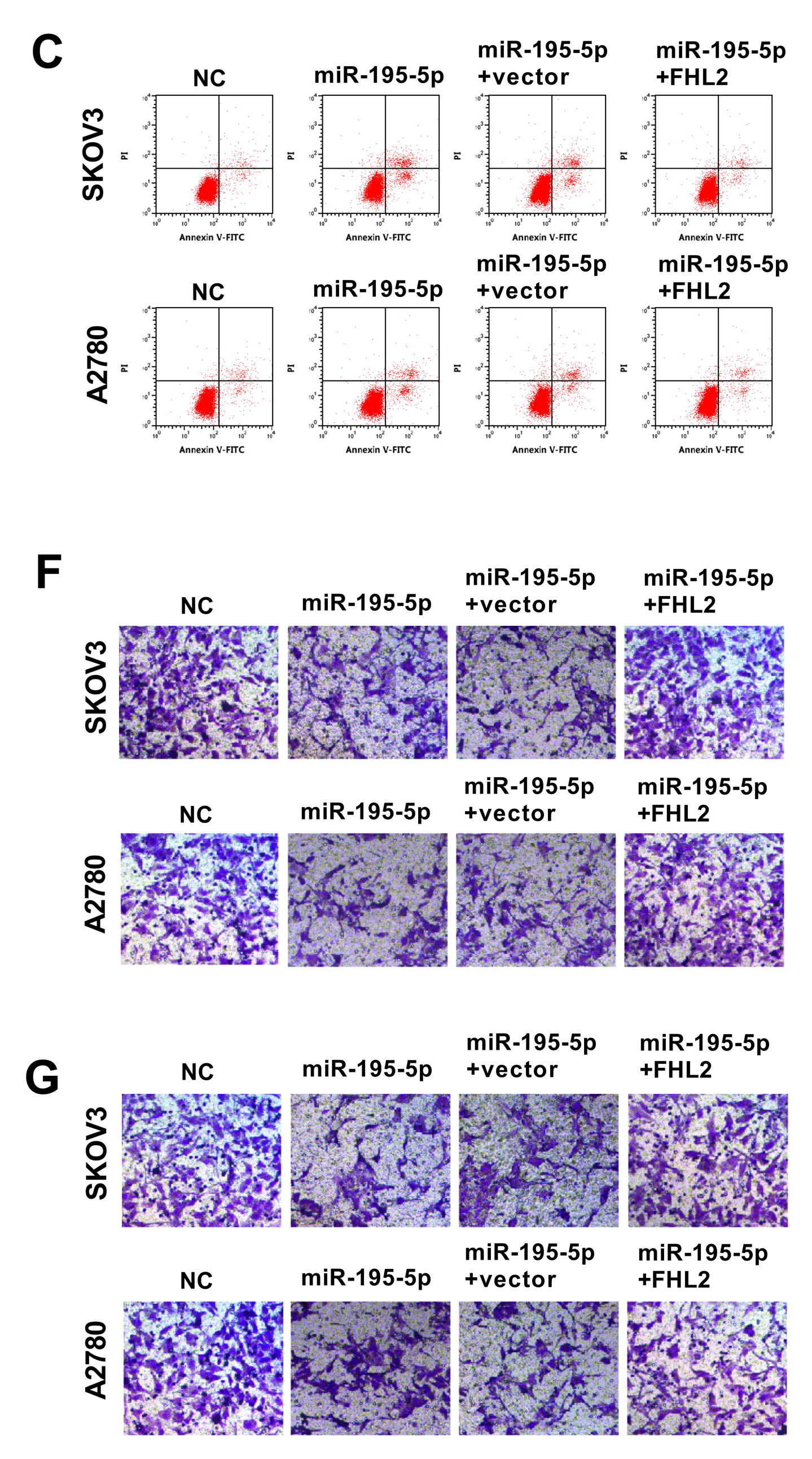

Supplement: Supplementary file 2 — Additional file 2: Figure S6 The representative images of (c) apoptosis, (f) migration and (g) invasion in SKOV3 and A2780 cells transfected with miR-195-5p, NC, miR-195-5p+FHL2 or miR-195-5p+vector. [file 12935_2020_1452_MOESM2_ESM.tif]
